# GC content strongly influences the role of poly(dA) in the intrinsic nucleosome positioning in *Saccharomyces cerevisiae*

**DOI:** 10.1002/yea.3701

**Published:** 2022-03-29

**Authors:** Edoardo Trotta

**Affiliations:** ^1^ Institute of Translational Pharmacology Consiglio Nazionale delle Ricerche (CNR) Rome Italy

**Keywords:** GC content, nucleosome occupancy, nucleosome positioning, poly(dA)

## Abstract

The nucleosome is the basic structural element of genomic DNA packaging and plays a role in transcription, replication, and recombination. Poly(dA) tracts are considered major sequence determinants of nucleosome positioning, although their role is not well understood. Here, we show that the homopolymeric character and the low GC content of poly(dA)s play different roles in nucleosome formation. We found that the inherent low GC content of poly(dA) alone can account for the deep and anisotropic nucleosome depletion at structurally and functionally important regions of promoters and origins of replication. We also show that the level of nucleosome occupancy at poly(dA) is strongly related to the local nucleotide background and its high frequency of occurrence in *Saccharomyces cerevisiae* does not appear merely to be associated with its intrinsic nucleosome‐excluding properties. In addition, we show that the GC content alone can predict more than 60% of the in vitro nucleosome map, providing further evidence that the intrinsic nucleosome positioning is more greatly determined by GC content than poly(dA) stretches. Our results are consistent with a model in which poly(dA) stretches act at two distinct levels: first, by its low GC content, which intrinsically contributes to hinder nucleosome formation, and second, by its contiguous runs of dA that selectively drive the recruitment of non‐histone proteins with structural and functional roles.

## Take‐away

- The homopolymeric nature and the GC content of poly(dA)s play different roles in the *Saccharomyces cerevisiae* genome.
- GC content explains the intrinsic asymmetric low nucleosome occupancy at poly(dA) sites.
- The high frequency of poly(dA) is not simply related to its intrinsic nucleosome‐excluding property.
- GC content can predict more than 60% of in vitro nucleosome occupancy.
- The nucleosome occupancy of AAAAA is strongly dependent on the background nucleotide composition.

## Graphical abstract

The cross‐correlation of dA, dT, and GC content with nucleosome occupancy in autonomously replicating sequences of in vitro reconstituted chromatin. The inherent low GC content (high AT content) of poly(dA) alone can account for the deep and anisotropic nucleosome depletion at structurally and functionally important regions of promoters and origins of replication in *Saccharomyces cerevisiae*.

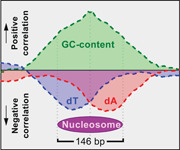

## 1 INTRODUCTION

The nucleosome is a DNA–protein complex forming the primary structural element of chromatin. A nucleosome particle contains a DNA segment of about 146 base pairs wrapped around an octamer core of histone proteins in a left‐handed superhelical winding (Luger et al., 1997). In *Saccharomyces cerevisiae,* about 81% of chromosomal DNA is wrapped in nucleosomes (Lee et al., 2007) that are connected by a DNA linker with a median length of 23 bp (for linkers ≤ 100 bp) (Brogaard et al., 2012). The nucleosome is not only the fundamental structural element of DNA packaging but also plays a role in DNA replication (Eaton et al., 2010; Groth et al., 2007), recombination (Bevington & Boyes, 2013), and transcription (Bai & Morozov, 2010; Han & Grunstein, 1988; Lee et al., 2007; Lorch et al., 1987) by modulating the accessibility of regulatory proteins to chromosomes. DNA sequence, transcription factors, chromatin remodelers, and the PolII transcriptional machinery are determinant factors of nucleosome positioning (Segal & Widom, 2009b; Struhl & Segal, 2013). Of these, the DNA sequence plays a primary role (Kaplan et al., 2009) and can account for at least 50% of the in vivo nucleosome arrangement (Segal et al., 2006). The two major DNA determinants of nucleosome positioning are the GC content and poly(dA) stretches. The GC content is positively correlated with nucleosome occupancy (Tillo & Hughes, 2009), whereas poly(dA) tracts are associated with the nucleosome‐depleted regions (NDRs) of promoters (Wu & Li, 2010) and autonomously replicating sequences (ARSs) (Eaton et al., 2010; Field et al., 2008).

Poly(dA) tracts are enriched in eukaryotic genomes (Dechering et al., 1998) and are identified as dominant intrinsic determinants of nucleosome organization both in vivo‐ and in vitro‐reconstituted chromatin (Chereji & Clark, 2018; Segal & Widom, 2009a). The poly(dA) sequences have nucleosome‐excluding properties, which are believed to be caused by their rigid structure that may hinder DNA wrapping around histone octamers (Segal & Widom, 2009a; Suter et al., 2000). The magnitude of the nucleosome‐excluding properties of poly(dA) sequences increases with their length (Kunkel & Martinson, 1981; Prunell, 1982), and a large fold depletion was also found in poly(dA) tracts with several base–pair substitutions (Field et al., 2008). However, the nucleosome‐excluding properties of poly(dA) tracts are not absolute and they can be incorporated into the nucleosome (Bao et al., 2006; Getts & Behe, 1992; Losa et al., 1990; Mahloogi & Behe, 1997; Prunell, 1982; Puhl & Behe, 1995). A peculiar characteristic of in vivo poly(dA) sequences is that they can be positioned asymmetrically downstream with respect to the NDRs, constituting a possible barrier to nucleosome movement (de Boer & Hughes, 2014; Wu & Li, 2010). The asymmetry of nucleosome depletion at poly(dA) tracts of in vitro‐reconstituted chromatin is controversial. Indeed, it has been reported that this asymmetry is absent in chromosomes reconstituted in the presence of whole‐cell extract, although it is restored after ATP addition that triggers chromatin remodelers (de Boer & Hughes, 2014). On the other hand, additional studies found the asymmetric nucleosome depletion at poly(dA) tracts of ARS sites in nucleosomes assembled in vitro using only purified histones and DNA (Eaton et al., 2010).

In the present work, we investigated the relationship between DNA sequence and nucleosome occupancy in chromatin assembled in vitro with purified histones and DNA (Kaplan et al., 2009). Our results suggest that the GC content is the dominant factor of the intrinsic nucleosome positioning, explaining at least 60% of the in vitro nucleosome occupancy. Remarkably, the intrinsic role of poly(dA) in the nucleosome positioning appears to be only correlated with its inherently low GC content and not with its homopolymeric trait. Our results are consistent with a model in which the GC content intrinsically affects the nucleosome formation, whereas the homopolymeric nature of poly(dA) acts indirectly by driving chromatin remodelers to slide or destabilize nucleosomes.

## 2 MATERIALS AND METHODS

### 2.1 Genome‐wide data sets

The genome annotation of *S. cerevisiae* S288C, which includes the genomic coordinates of ARSs and coding DNA sequences (CDSs), was the release R64 (saccharomyces_cerevisiae_R64‐2‐1_20150113.gff) downloaded from the Saccharomyces Genome Database (SGD) website (http://www.yeastgenome.org). The processed genome‐wide data of in vivo nucleosome occupancy mapped by micrococcal nuclease (MNase)‐seq (accession number GSM3304656, file: GSM3304656_screen_wtm_cov.wig.bw) (Klein‐Brill et al., 2019) and H3 chemical cleavage (accession number GSM2561057, file: GSM2561057_Occupancy_H3_CC_rep_1.bw) (Chereji et al., 2018) were taken from the Gene Expression Omnibus (GEO) (https://www.ncbi.nlm.nih.gov/geo). Genome‐wide maps of nucleosome occupancy of in vitro‐assembled chromosomes (Kaplan et al., 2009; Zhang et al., 2009) were downloaded from the SGD archive and GEO (accession number GSM379239, file: GSM379239_SALT_yeast_146bp.wig), respectively. 5′ and 3′ ends of quantified transcript isoforms (TIFs) were taken from the Supplementary Data 1 of the work of Pelechano et al. (2013). The coordinates of transcription start sites (TSSs) to define the 200 nt promoter regions and the surrounding average nucleosome occupancy were estimated by the median of the 5′ UTR length of major TIFs reported in Supplementary Data 3 of the work of Pelechano et al. (2013). Binary data in bigWig format (.bw) were converted into the bedGraph text format using the program bigWigToBedgraph (http://hgdownload.cse.ucsc.edu/admin/exe/). Wiggle (.wig)‐type files were converted into bedGraph using a simple format converter written in C# in our laboratory.

### 2.2 Data processing and analysis

Data were processed using software programs written in our laboratory in C#, which were tested using the statistical tools of the software environment R (https://www.r-project.org). Cross‐ and auto‐correlation analyses were performed by computing the Spearman's rank (*Rs*) or Pearson's correlation coefficient (*Rp*) between two aligned genome‐wide profiles progressively shifted by 1 bp, typically from −600 to +600 bp (Figure S1). Genome‐wide *Rs* and *Rp* cross‐correlations between nucleosome occupancy and the density of 5′ end of TIFs were performed using our software programs YeastpRofileSpearman (https://sourceforge.net/projects/yeastprofilespearman/) and YeastpRofile (https://sourceforge.net/projects/yeastprofile/), respectively. When cross‐correlation was performed on only a specified genomic category, as CDSs, promoters, or ARSs, the regions outside the category boundaries of the first profile were excluded. The analyses of *Rp* cross‐correlations (Figure S2) were qualitatively similar enough to the *Rs* cross‐correlations; therefore, here, we report only those related to *Rs*.

The statistically expected frequencies of poly(dA)s in ARSs, promoters, and CDSs were estimated from the native sequences after shuffling their nucleotides within each individual genomic feature. The average frequency of 100 shufflings was used in calculations.

For the cross‐correlation of nucleotide occurrences, the genomic nucleotide sequence was converted into a numerical binary sequence by replacing the specific nucleotide with 1 and the others with 0.

Genome‐wide DNA structural features were predicted using the DNAshape method (Zhou et al., 2013).

### 2.3 Prediction of nucleosome occupancy

The −100 to +100 nt region of the GC content versus nucleosome occupancy cross‐correlation profile was used to predict the genomic nucleosome occupancy. To this end, a centered sliding window of 201 nucleotides was used to assign a score to each nucleotide in the genomic sequence. At every single nucleotide of the 201 genomic positions within the sliding window, we assigned the *Rs* value of the corresponding position in the cross‐correlation profile if it was dG or dC; otherwise, we assigned 0 if the nucleotide was dA or dT. Finally, the average of the 201 *Rs* values was assigned at the nucleotide in the center of the genomic sliding window. When we added dA and dT scores in the procedure to predict nucleosome occupancy by GC content, the *Rs* values of dA and dT profiles were assigned to the respective bases of the genomic sliding window.

## 3 RESULTS

### 3.1 Cross‐correlation profile of nucleosome occupancy relative to the TSSs

Cross‐correlation measures the variation of the correlation between two signal profiles as a function of the displacement of one relative to the other. It can identify the presence and the distance of possible correlated patterns that are systematically shifted between the two profiles. In this study, we cross‐correlated the genome‐wide profiles of nucleosome occupancy with various structural and functional profiles of the genomic sequence.

The genome‐wide profile of nucleosome occupancy can be achieved using various technical procedures, including the most popular digestion of chromatin with MNase (Noll, 1974) and the chemical mapping using targeted mutations in histones (Brogaard et al., 2012; Chereji et al., 2018). We used both sources of nucleosome occupancy data to check if possible inherent artifacts of the mapping method could alter the cross‐correlation results, even if nucleosome mapping is not substantially biased by MNase (Allan et al., 2012).

**Figure 1:**
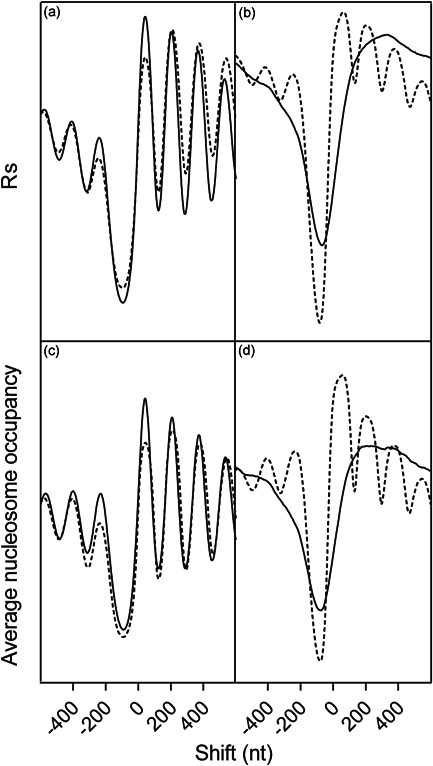
(a) and (b) Genome‐wide cross‐correlograms of nucleosome occupancy with the density of 5′ end of TIFs. (c) and (d) Nucleosome genome‐wide average occupancy around TSSs. (a) and (c) Profiles of in vivo nucleosome occupancy mapped by MNase (Klein‐Brill et al., 2019) (solid line) and by chemical cleavage (Chereji et al., 2018) (dashed line). (b) and (d) Profiles of nucleosome occupancy of in vivo (dashed line) and in vitro‐reassembled (solid line) chromatin mapped by MNase (Kaplan et al., 2009). TIF, transcript isoform; TSS, transcription start site

We first evaluated the capability of the cross‐correlation analysis to obtain an unbiased profile of the well‐known nucleosome arrangement around the TSS (Lieleg et al., 2015). Figure 1a,b show the cross‐correlograms of the quantitative map of the 5′‐end of TIFs (Pelechano et al., 2013) with in vivo and in vitro nucleosome occupancy profiles determined by MNase (Kaplan et al., 2009; Klein‐Brill et al., 2019) and chemical (Chereji et al., 2018) mapping procedures. For comparison, Figure 1c,d show the average nucleosome occupancy around TSSs. As shown in the cross‐correlation profiles of Figure 1, the previously well‐established in vitro and in vivo nucleosome patterns around the TSS (Kaplan et al., 2009; Lieleg et al., 2015) are well reproduced. Indeed, all correlograms show the canonically positioned deep NDR at promoters just upstream of the TSS. Additionally, the cross‐correlograms of in vivo nucleosome occupancy also show the typical periodic nucleosome array downstream of the TSS that is not present in the in vitro nucleosome map (Figure 1b). Finally, Figure 1 also shows an acceptable similarity among the cross‐correlogram profiles of the differently acquired data sets of in vivo nucleosome occupancy, consistent with the absence of specific artifacts associated with the MNase procedure. It should be noted that the cross‐correlation analysis has the advantage that it considers the 5′‐end of all TIFs and not a simple point estimator of the TSSs, such as the mean or median.

### 3.2 Cross‐correlation of nucleotide occurrences and GC content with nucleosome occupancy

**Figure 2:**
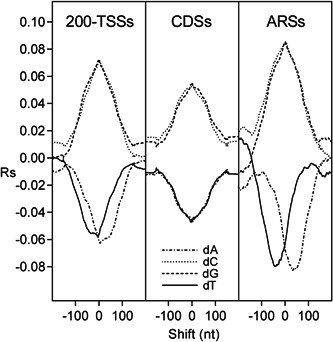
Cross‐correlograms of the four bases dA (dash‐dotted line), dC (dotted line), dG (dashed line), and dT (solid line) with nucleosome occupancy in 200‐TSSs, CDSs, and ARSs. ARS, autonomously replicating sequence; CDS, coding DNA sequence; TSS, transcription start site

**Figure 3:**
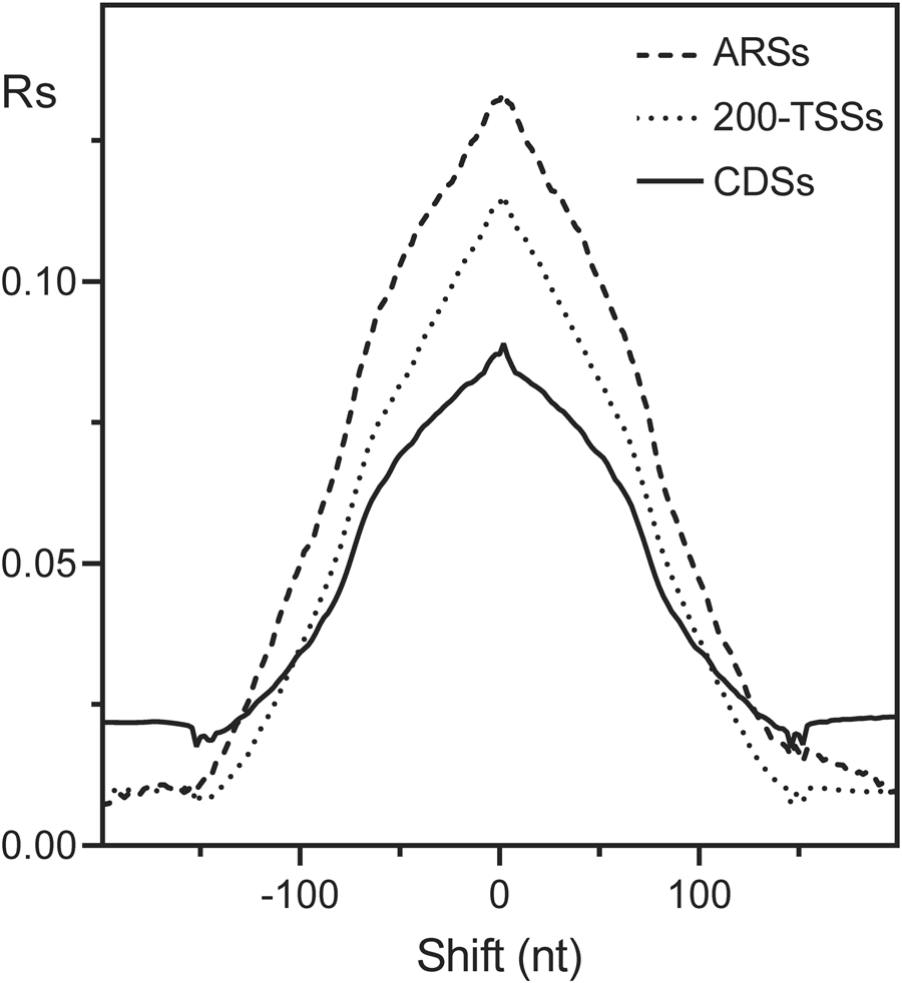
Cross‐correlograms between GC content and nucleosome occupancy in 200‐TSSs (dotted line), CDSs (solid line), and ARSs (dashed line). ARS, autonomously replicating sequence; CDS, coding DNA sequence; TSS, transcription start site

To clarify the intrinsic role of poly(dA) and GC content in the genomic arrangement of nucleosomes, we cross‐correlated the occurrence of the four nucleotides and GC content with the nucleosome occupancy of chromatin assembled in vitro from purified DNA and histone octamers (Kaplan et al., 2009). Since poly(dA) has been previously associated with NDRs of promoters (Mavrich et al., 2008; Struhl, 1985) and ARSs (Eaton et al., 2010), we performed the cross‐correlation analysis separately for ARSs, promoters, and CDSs. The analyzed promoter region was the fragment containing 200 nt upstream of the TSS, which we termed 200‐TSS. As shown in the cross‐correlograms of Figure 2, in ARSs and 200‐TSSs, the most negative peak of dA (dT) plot is downstream‐shifted (upstream‐shifted) with respect to the nucleosome depletion, in agreement with an in vitro asymmetric positioning of poly(dA) (poly(dT)) at the NDRs. Conversely, in CDSs, the most negative correlation of dA (dT) with nucleosome occupancy is symmetric and at zero shift, consistent with an isotropic depletion of the nucleosome at poly(dA) (poly(dT)) tracts of CDSs. In contrast to dA and dT, GC content shows an isotropic positional relationship with nucleosomes also in ARSs and 200‐TSSs (Figure 3). It should be noted that the increasing positive correlation between GC content and nucleosome occupancy toward the nucleosome dyad axis (Figure 3) can explain why the mononucleosome DNA of longer MNase chromatin digestions is relatively richer in GC (Chereji et al., 2017).

**Figure 4:**
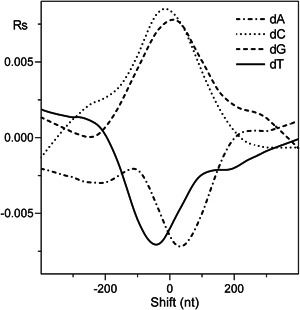
Cross‐correlograms of the four bases dA (dash‐dotted line), dC (dotted line), dG (dashed line), and dT (solid line) with GC content. The plots were smoothed using *LOWESS* (locally weighted scatter plot smoothing) curve fitting (Chambers et al., 1983)

Finally, Figure 4 shows the smoothed cross‐correlograms of the four nucleotides with GC content. As shown, dA and dT show an asymmetric positioning with respect to GC content, similar to that shown with respect to nucleosome occupancy (Figure 2), consistent with a possible role of GC content in the anisotropic positioning of dA and dT at the nucleosome‐depleted sites of ARSs.

### 3.3 Cross‐correlation between nucleotide occurrences

**Figure 5:**
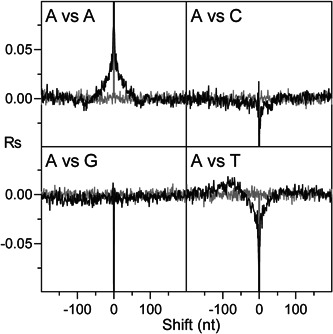
Auto‐correlogram of dA occurrence and cross‐correlograms of dA with dC, dG, and dT in native (black lines) and in shuffled (gray lines) ARS sequences. ARS, autonomously replicating sequence

To investigate the reciprocal positioning of the four nucleotides, we cross‐correlated their occurrences in CDSs, ARSs, and 200‐TSSs. The results showed that in ARSs and 200‐TSSs, the occurrence of dAs (dTs) is positively auto‐correlated within about 50 nt (Figure 5). This result agrees with our finding that poly(dA)s and poly(dT)s in ARSs and 200‐TSSs are statistically more frequent than expected from the local nucleotide composition (see below). We also found that in ARSs and 200‐TSSs, but not in CDSs, the occurrence of dA (dT) is positively correlated with the occurrence of dT (dA) in a region located upstream (downstream) at about 70–80 nt (Figure 5), consistent with the positioning of poly(dA) at both strands of some depleted tracts. Surprisingly, in ARSs and 200‐TSSs, the occurrences of dA and dT are negatively correlated with each other within about 50 nt (Figure 5). This result is unexpected because if the enrichment of poly(dA)s in ARSs and 200‐TSSs is due to their intrinsic nucleosome‐excluding property, then it is unclear why dT is less favored than dG and dC. Finally, the expected 3‐base periodicity (Trifonov & Sussman, 1980) characterizes the cross‐ and auto‐correlograms of the four nucleotides in CDSs (Figure S3).

### 3.4 Prediction of the genome‐wide nucleosome density profile

**Figure 6:**
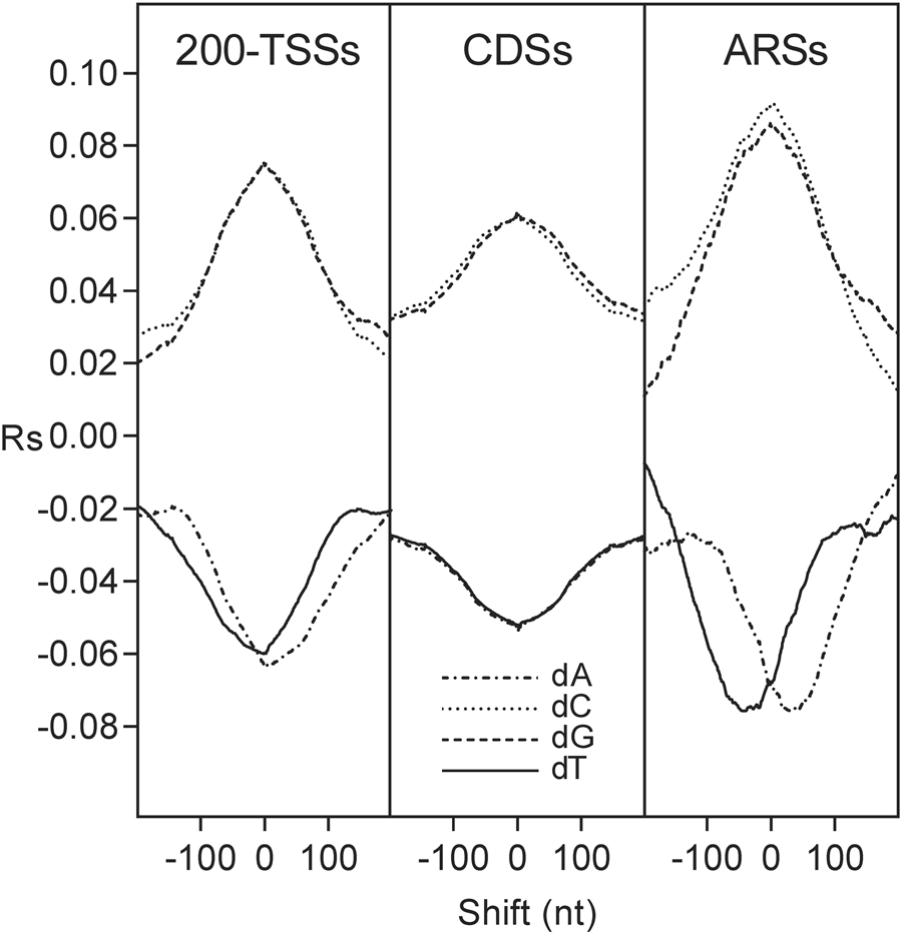
Cross‐correlograms of dA (dash‐dotted line), dC (dotted line), dG (dashed line), and dT (solid line) occurrence with the nucleosome occupancy predicted by GC content in 200‐TSSs, CDSs, and ARSs. ARS, autonomously replicating sequence; CDS, coding DNA sequence; TSS, transcription start site

We analyzed the genome‐wide nucleosome occupancy predicted using only GC content to establish if the nucleosome preference for GC‐rich sequences is enough to explain the anisotropic nucleosome depletion at poly(dA) sites. Above, we determined the cross‐correlation profile of GC content with nucleosome occupancy. Here, we used the 201 nt central window of that cross‐correlation profile to predict the genome‐wide nucleosome occupancy, as described in the Materials and Methods section. Surprisingly, the nucleosome occupancy predicted by GC content alone shows the same asymmetric cross‐correlation profile with dA and dT that we found at ARS and 200‐TSS sites of reassembled chromatin, as well as the symmetric cross‐correlation profiles at CDSs (Figure 6). Moreover, the simulated nucleosome occupancy also shows the canonical deep depletion upstream of the TSS, even if it is slightly shifted with respect to the reconstituted chromatin (Figure S5). We also found a strong correlation between the simulated and the in vitro genome‐wide nucleosome occupancy (*Rp* = 0.78), indicating that GC content alone can explain more than 60% of the intrinsic nucleosome occupancy in *S. cerevisiae* (coefficient of determination *R* ^2^ = 0.61) (Figure S4).

Finally, when we additionally included the profiles of dT and dA in the calculations of the simulated nucleosome occupancy by GC content, we did not observe any improvement in the prediction accuracy.

### 3.5 Cross‐correlation of nucleotide occurrences and GC content with the transcription start and termination sites (TSSs and TTSs)

**Figure 7:**
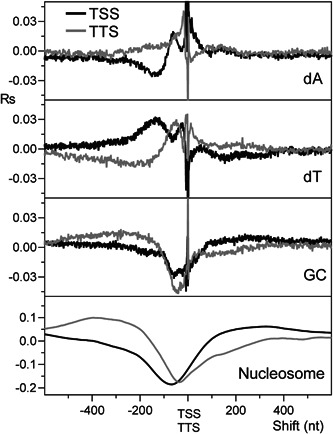
Cross‐correlograms of dA, dT, GC content, and nucleosome occupancy with the quantified TSS and TTS of the transcript isoforms. TSS, transcription start site; TTS, transcription termination site

To clarify the role of DNA sequence in the formation of NDRs, we compared the DNA sequence and the nucleosome occupancy of the regions upstream of the TSSs and TTSs. For this purpose, we computed the cross‐correlation profile of the four nucleotides, GC content, and nucleosome occupancy with the quantified TSS and TTS of the transcript isoforms (Pelechano et al., 2013) (Figure 7). As shown in Figure 7, the cross‐correlograms of GC content are consistent with those of nucleosome occupancy at both TSS and TTS, in agreement with a widespread inhibitory effect of low GC content on nucleosome formation. In contrast, the cross‐correlation profiles of dA or dT at the depleted sites of TSS are not consistent with those of TTS, indicating the lack of a possible common mechanism of poly(dA) in favoring the nucleosome depletion upstream of the two transcription sites.

### 3.6 Poly(dA)

**Figure 8:**
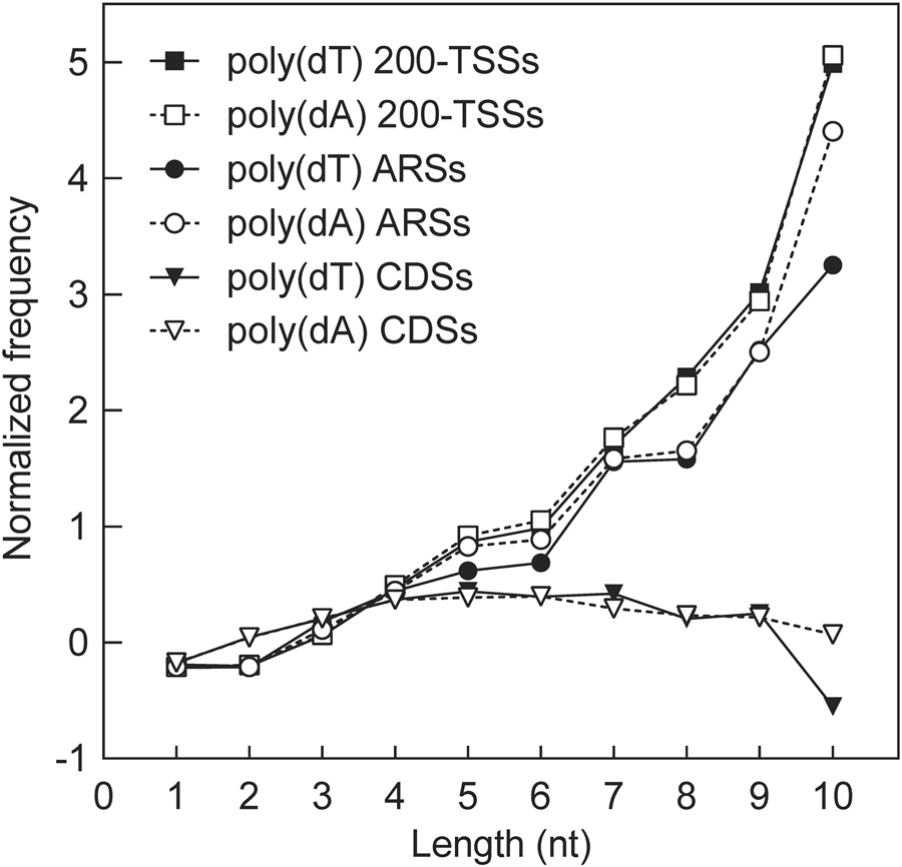
Normalized frequencies of various exact‐length homopolymeric tracts of dT (solid lines) and dA (dashed lines) in CDSs (triangles), ARSs (circles), and 200‐TSSs (squares). The frequencies were normalized by the log2 ratio with their expected frequencies from sequence composition. ARS, autonomously replicating sequence; CDS, coding DNA sequence; TSS, transcription start site

We analyzed the frequencies of occurrence and the nucleosome occupancy of poly(dA)s in CDSs, 200‐TSSs, and ARSs. Figure 8 shows the frequencies of poly(dA)s in the three genomic categories after normalization by the log2 ratio with their expected frequencies based on sequence composition (see Section 2 for details). As shown, poly(dA) and poly(dT) stretches longer than 4 nt show a much higher frequency than expected in ARSs and 200‐TSSs.

**Figure 9:**
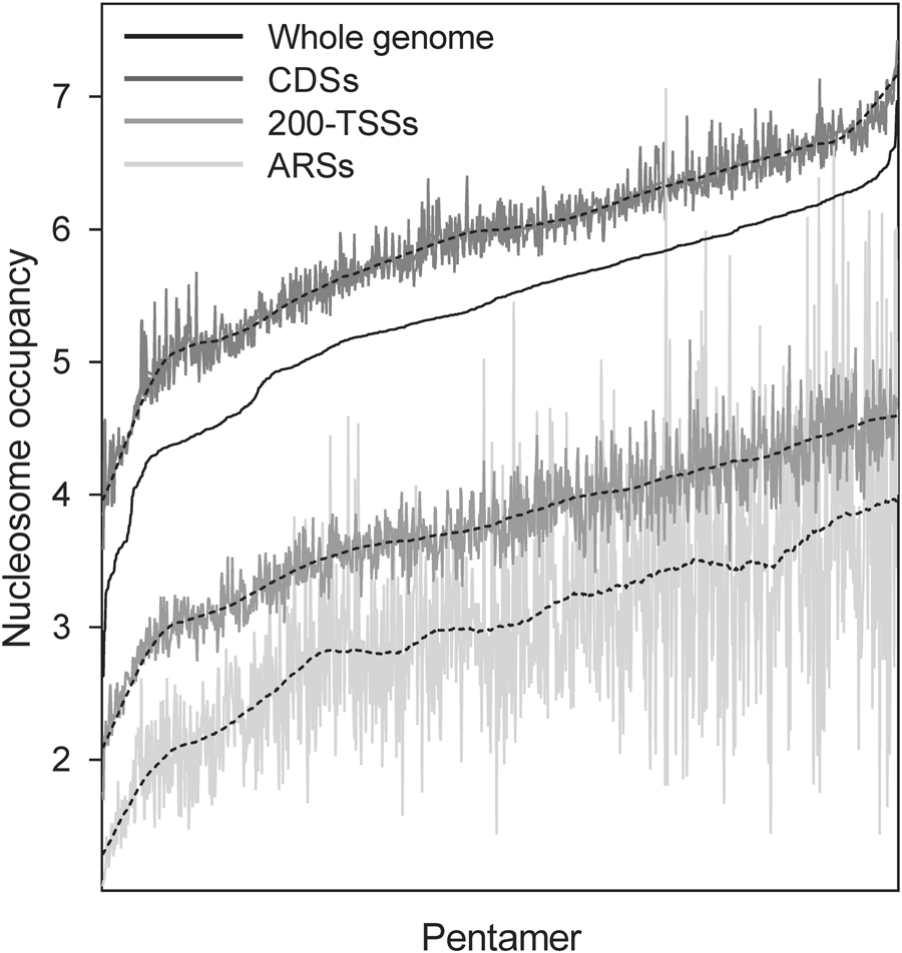
Average nucleosome occupancy of the 1024 pentamers in the whole genome (black line), in CDSs (dark gray line), in 200‐TSSs (gray line), and in ARSs (light gray line). The pentameric sequences are rank‐ordered along the *x* axis according to their genome‐wide average occupancy. The dashed black lines indicate the smoothed trends of the nucleosome occupancy of pentamers in CDSs, 200‐TSSs, and ARSs. ARS, autonomously replicating sequence; CDS, coding DNA sequence; TSS, transcription start site

Figure 9 shows the average nucleosome occupancy in ARSs, 200‐TSSs, and CDSs of the 1024 pentameric sequences that are rank‐ordered along the *x*‐axis according to their average nucleosome occupancy in the whole genome (black line of Figure 9). As shown in Figure 9, the nucleosome occupancy of pentamers is generally higher in CDSs than in 200‐TSSs and ARSs, consistent with their differences in GC content (40%, 36%, and 31%, respectively). Remarkably, the average nucleosome occupancy of AAAAA in CDSs is higher than the average occupancy of 812 (79%) pentamers in ARSs, reflecting the strong influence of the background nucleotide composition on the intrinsic nucleosome formation. Notably, after AAAAA and TTTTT, the pentamers with the lowest average nucleosome occupancy were ATATA and TATAT. However, in contrast with AAAAA and TTTTT, ATATA and TATAT present a frequency of occurrence in ARSs and 200‐TSSs lower than expected by nucleotide composition. This inconsistency between the frequency and the nucleosome occupancy of pentamers suggests that the nucleosome‐excluding property of AAAAA might not be the determinant factor of its high frequency in ARSs and 200‐TSSs. Consistent with the lack of relationship between frequency and nucleosome occupancy of poly(dA)s, we also found that in ARSs and 200‐TSSs, the dinucleotides AA and TT show low average nucleosome occupancy and high frequency, whereas the AT and TA dimers show low nucleosome occupancy, but lower frequency than expected.

**Figure 10:**
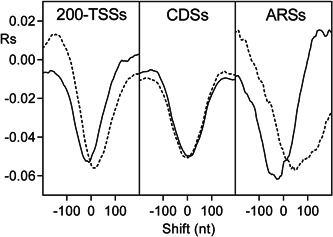
Cross‐correlograms of the occurrence of exact pentamers AAAAA (dashed lines) and TTTTT (solid lines) with in vitro nucleosome occupancy in ARSs, CDSs, and 200‐TSSs. ARS, autonomously replicating sequence; CDS, coding DNA sequence; TSS, transcription start site

Finally, we also cross‐correlated the occurrence of exact pentamers AAAAA and TTTTT with in vitro nucleosome occupancy in ARSs, CDSs, and 200‐TSSs, and the results confirm that the nucleosome depletion is asymmetrically positioned relative to AAAAA and TTTTT in ARSs and 200‐TSSs, but not in CDSs (Figure 10).

### 3.7 Relationship between nucleosome occupancy and GC content

**Figure 11:**
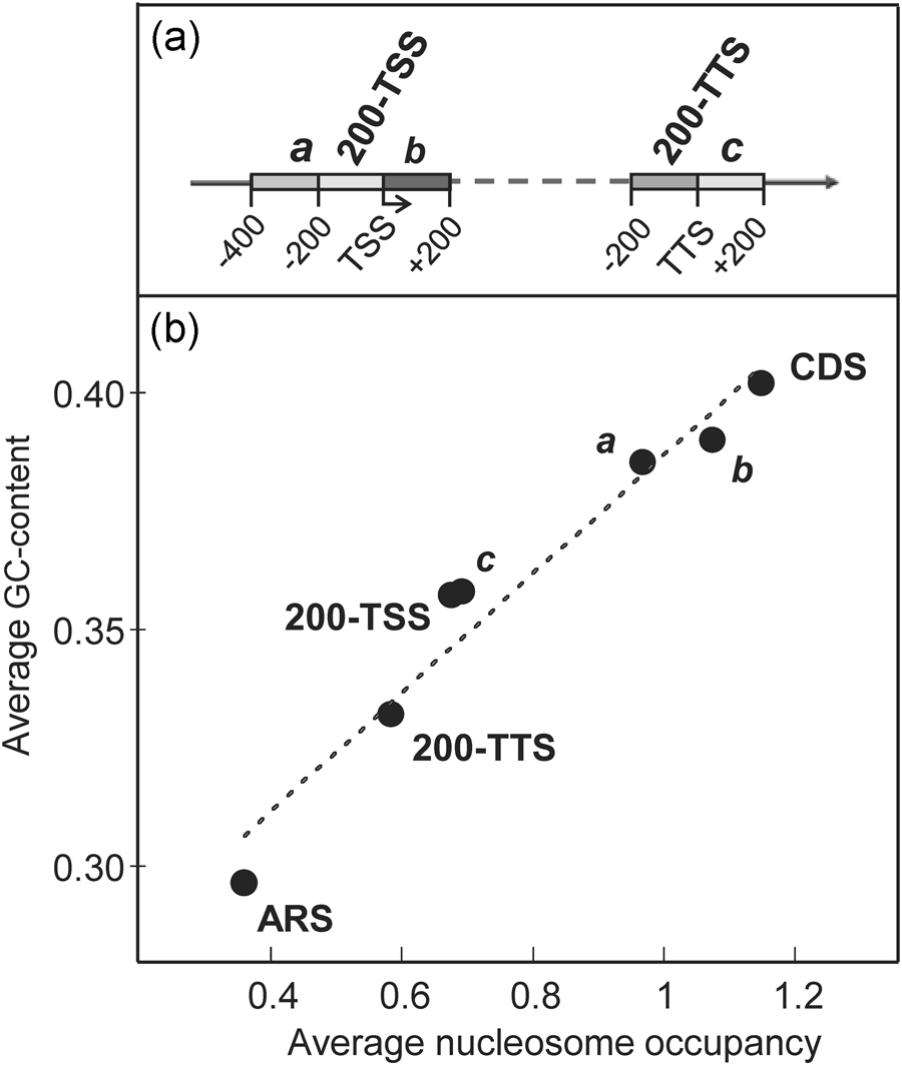
(a) Schematic representation of the regions located near the TSS and TTS used to analyze the relationship between GC content and nucleosome occupancy. (b) Scatter plot of the relationship between the average GC content and nucleosome occupancy in ARSs, CDSs, and the regions around the TSS and TTS indicated in panel A. The broken line indicates linear regression. ARS, autonomously replicating sequence; CDS, coding DNA sequence; TSS, transcription start site; TTS, transcription termination site

We analyzed the relationship between nucleosome occupancy and GC content of different genomic regions to establish if the intrinsic low nucleosome occupancy of ARSs and 200‐TSSs can be explained by their GC content. The relationship between nucleosome occupancy and GC content, illustrated in Figure 11, shows that the low average nucleosome occupancy at ARSs and 200‐TSSs can be accurately estimated from their average GC content (*Rp* = 0.98).

### 3.8 Relationship between nucleosome occupancy and predicted structural features

To test if the anisotropic position of poly(dA) relative to nucleosome depletion reflects an anisotropy of the DNA structure, we cross‐correlated the in vitro nucleosome occupancy with the structural features predicted by the DNAshape method (Zhou et al., 2013). We found that nucleosome occupancy was negatively correlated with the helical twist of DNA and positively correlated with minor groove width, propeller twist, and roll of DNA. Consistent with the cross‐correlation profile of GC content with nucleosome occupancy, the results showed that at ARSs, 200‐TSSs, and CDSs, the cross‐correlation peaks of all structural features analyzed were symmetric with respect to the nucleosome occupancy.

## 4 DISCUSSION

We found that both ARSs and 200‐TSSs show an asymmetric positioning of poly(dA)s relative to nucleosome‐depleted tracts in in vitro‐assembled chromosomes. It is unclear how this in vitro asymmetric positioning is generated and if the enrichment of poly(dA) at 200‐TSS and ARS sites is determined by its nucleosome‐excluding property. Since two major characteristics of poly(dA) tracts are the low GC content and dA‐homopolymeric nature, we evaluated the hypothesis that these two sequence features could play distinct roles: the low GC content could be the primary determinant of the intrinsic nucleosome arrangement at poly(dA) sites including the asymmetric nucleosome depletion, whereas the dA‐homopolymeric trait may be the target of functional and structural non‐histone proteins. All our results were consistent with this hypothesis.

First, we found that the nucleosome occupancy simulated using only GC content can predict not only the in vitro genome‐wide occupancy with 60% accuracy (Figure S4) and the strong NDR at the promoters (Figure S5) but that it can also reproduce the native anisotropic nucleosome depletion at poly(dA) sites of ARSs and promoters together with the symmetric disposition at CDSs (Figure 6). In agreement with this result, we found that the nucleosome occupancy is perfectly aligned with the GC content in ARSs, 200‐TSSs, and CDSs, as shown by the strong positive peak at zero shift in the cross‐correlograms of in vitro reconstituted nucleosomes (Figure 3). Accordingly, we found that the cross‐correlation profiles of GC content and nucleosome occupancy relative to the TSS and TTS are very similar (Figure 7). We also found that the cross‐correlation profile of dA (or dT) with GC content (Figure 4) is very similar to that with nucleosome occupancy (Figure 2), suggesting that intrinsic positioning of dA and dT with respect to nucleosome arrangement could be mediated by GC content.

Our analyses also showed that the mere presence of a dA‐homopolymeric tract is not enough to generate the asymmetric nucleosome occupancy. Indeed, we found that the pentamers AAAAA and TTTTT are positioned asymmetrically with respect to nucleosome arrangement in ARSs and 200‐TSSs and symmetrically in CDSs (Figure 10).

Additionally, we found that the high frequency of occurrence of poly(dA)s of various lengths in ARSs and 200‐TSSs (Figure 8) does not appear to be related to their intrinsic nucleosome‐excluding property. In fact, we found that the pentamer ATATA, which shows very low nucleosome occupancy almost equal to AAAAA, does not show a frequency of occurrence at ARSs and 200‐TSSs higher than expected. Moreover, the average occupancy of ATATA is lower than TAAAA and AAAAT, showing that the AA dinucleotide does not disfavor nucleosome formation more than AT or TA. Also, in ARSs and 200‐TSSs, AT and TA dimers are less frequent than expected by sequence composition despite their low nucleosome occupancy. Against a causal relationship between low nucleosome occupancy and high frequency of poly(dA) in ARSs and 200‐TSSs, we also found that the occurrence of dT is less favored than dG and dC in the proximity of dA (Figure 5), consistent with a nucleotide preference that is not driven by the necessity to obtain the best nucleosome‐excluding sequence.

We also found that the average nucleosome occupancy of various genomic regions, including 200‐TSSs and ARSs, can be accurately predicted using linear regression with the GC content (*Rp* = 0.98) (Figure 11), which appears to be the leading determinant of nucleosome occupancy also in 200‐TSSs and ARSs. Indeed, consistent with a key role of nucleotide composition against runs of consecutive dAs in the nucleosome depletion, we found that the pentamer AAAAA in CDSs presents an average nucleosome occupancy higher than 79% of the 1024 different pentamers in ARSs (Figure 9), which is in agreement with the higher GC content of CDSs compared to ARSs. The role of GC content is also apparent when comparing the NDRs upstream of TSSs and TTSs. In those two transcriptional regions, dA and dT are differently positioned with respect to nucleosome occupancy, whereas there is a clear concomitance between GC content and nucleosome occupancy (Figure 7). Finally, we showed that structural features with a possible role in nucleosome positioning, including minor groove width, roll, propeller twist, and helix twist, are symmetrically arranged with respect to nucleosome occupancy in ARSs, 200‐TSSs, and CDSs, being more consistent with GC content than with poly(dA) stretches.

Previous studies also support the minor role of the homopolymeric nature of poly(dA) in the intrinsic nucleosome positioning and stability. For example, in mice and humans, which have histones very similar to yeast, the trend of biased nucleosome occupancy around poly(dA) and poly(dT) is opposite (de Boer & Hughes, 2014). In *Schizosaccharomyces pombe*, poly(dA) tracts affect, but do not deplete, nucleosomes (Moyle‐Heyrman et al., 2013) and are not enriched at NDRs (Lantermann et al., 2010). In addition, a study considering 12 Hemiascomycota yeast species reports that poly(dA) tracts can explain only 8.6%–25.7% of the nucleosome depletion at NDRs within a given species, and much of the depletion remains unexplained, for example, the highly expressed proteasomal genes that present deep NDR in all species, but are not associated with poly(dA) (Tsankov et al., 2010). Moreover, consistent with our conclusions about a limited role of the homopolymeric nature of poly(dA) in the intrinsic formation of NDRs at ARSs and promoters, a recent study measuring the DNA–histone affinities for 47 DNA sequences reported that the GC content significantly impacts the nucleosome formation, while poly(dA) tracts do not (Schnepf et al., 2020).

While the homopolymeric characteristic of poly(dA) seems to be barely relevant compared to GC content in in vitro nucleosome formation, the role of poly(dA) turns out to be more important in in vivo nucleosome arrangement. Indeed, poly(dA)s form an asymmetric barrier to the nucleosome transit mediated by ATP‐dependent chromatin remodelers (de Boer & Hughes, 2014). Additionally, poly(dA) tracts can interfere with the sliding of nucleosome by the Chd1 remodeler (Winger & Bowman, 2017), and the RSC chromatin remodeling complex recognizes poly(dA) direction and directionally displaces the nucleosome and produces NDRs at promoters (Krietenstein et al., 2016; Lorch et al., 2014). In addition, the origin recognition complex, which marks the site for the initiation of replication, recognizes the dT‐rich ARS consensus sequence and is required for the precise positioning of nucleosomes neighboring the origins of replication (Eaton et al., 2010).

In conclusion, our findings provide evidence consistent with a model in which poly(dA) contributes to the intrinsic nucleosome depletion due to its inherent low GC content and not its homopolymeric nature. In ARSs and promoters, the asymmetric positioning of poly(dA) stretches relative to the NDRs reflects its asymmetric positioning relative to regions with low GC content. The frequent occurrence of dA‐homopolymeric stretches in promoters and ARSs could reflect their double role of disfavoring nucleosome formation by their poor GC content and constituting a directional signal for non‐histone proteins implicated in both the stabilization of nucleosome‐free regions and the regulation of DNA transcription or replication.

## CONFLICT OF INTEREST

The author declares no conflict of interest.

## Supporting information

Supporting information.
