# Supplementary material for: GC content strongly influences the role of poly(dA) in the intrinsic nucleosome positioning in Saccharomyces cerevisiae
Source: Yeast. 2022 Mar 29;39(4):262–71. doi: 10.1002/yea.3701 (PMC9541940; doi:10.1002/yea.3701)
Supplement: Supplementary file 1 — Supporting information. [file YEA-39-262-s001.docx]

**Supplemental Material**

**
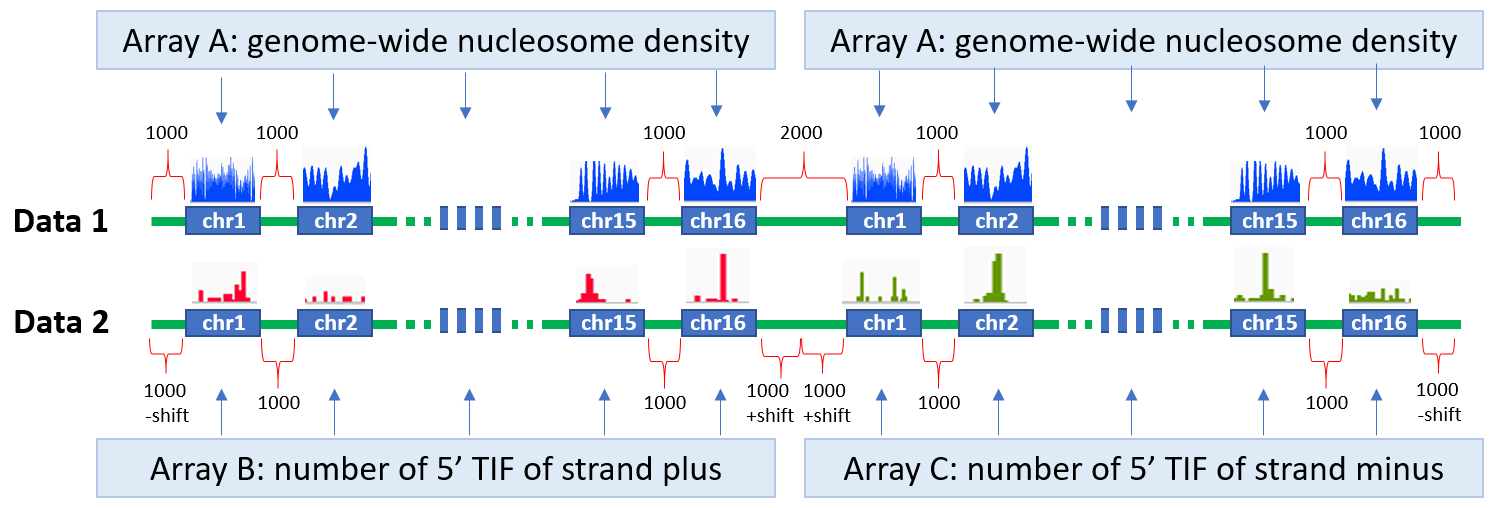
**

**Fig. S1**  Schematic representation of data alignments for cross-correlation analysis of Fig. 1. The numbers in the scheme indicate the number of null points added at the extremities of chromosomes to generate Data 1 and Data 2. In the cross-correlation analyses of Fig.1 shift was changed from 600 to -600 by step of 1 nt. The correlation coefficient between Data 1 and Data 2 was computed at each shift step.

**Fig. S2**  Genome-wide Pearson cross-correlograms of nucleosome occupancy with the log-transformed density of 5’ end of TIFs. A) profiles of in vivo nucleosome occupancy mapped by MNase (Klein-Brill et al., 2019) (solid line) and by chemical cleavage (Chereji et al., 2018) (dashed line). B) profiles of nucleosome occupancy of in vivo (dashed line) and in vitro-reassembled (solid line) chromatin mapped by MNase (Kaplan et al., 2009).

**Fig. S3**  Auto-correlogram of dA occurrence and cross-correlograms of dA with dC, dG and dT in native (blue lines) and in shuffled (red lines) CDS sequences. The enlarged view of the inset shows the three-base periodicity that characterizes CDSs.

**Fig. S4** Scatter plot comparing the simulated and the in vitro native nucleosome occupancy of nucleotides in S. cerevisiae genome. Nucleosome occupancy was predicted based on GC-content. Native in vitro nucleosome occupancy was taken from Kaplan et al. (2009). The broken line indicates linear regression.

**Fig. S5**  Genome-wide cross-correlograms of the nucleosome occupancy of in vitro reconstituted chromatin (blue line) and simulated nucleosome occupancy (red line) with the density of 5’ end of transcription isoforms. Nucleosome occupancy was predicted based on GC-content. Native in vitro nucleosome occupancy was taken from Kaplan et al. (2009).
